# A mixed methods approach to determine the climate of interprofessional education among medical and health sciences students

**DOI:** 10.1186/s12909-021-02645-4

**Published:** 2021-04-10

**Authors:** Nabil Sulaiman, Youssef Rishmawy, Amal Hussein, Maha Saber-Ayad, Hamzah Alzubaidi, Sausan Al Kawas, Hayder Hasan, Salman Y. Guraya

**Affiliations:** 1 Sharjah Institute for Medical Research, University of Sharjah, Sharjah, United Arab Emirates; 2 Department of Family & Community Medicine & Behavioral Sciences, College of Medicine, University of Sharjah, Sharjah, United Arab Emirates; 3 Baker Heart and Diabetes Institute, Melbourne, Victoria Australia; 4 Department of Clinical Sciences, College of Medicine & Research Institute for Medical and Health Sciences, University of Sharjah, Sharjah, United Arab Emirates; 5 College of Medicine, Cairo University, Giza, Egypt; 6 Pharmacy Practice & Pharmacotherapeutics, College of Pharmacy, University of Sharjah, Sharjah, United Arab Emirates; 7 Department of Oral & Craniofacial Health Sciences, College of Dental Medicine, University of Sharjah, Sharjah, United Arab Emirates; 8 Clinical Nutrition and Dietetics, College of Health Sciences, University of Sharjah, Sharjah, United Arab Emirates; 9 College of Medicine, University of Sharjah, Sharjah, United Arab Emirates

**Keywords:** IPE, Interprofessional education, Medical education, Multiprofessional, Learning, Gulf, Middle east, RIPLS, Focus groups

## Abstract

**Background:**

High-quality patient care is a complex phenomenon that requires collaboration among healthcare professionals. Research has shown that Interprofessional Education (IPE) carries promise to improve collaborative work and patient care. So far, collaboration among various health professionals remains a challenge. Very few focus group discussions to determine the medical students’ readiness and positive attitudes towards IPE have been reported from the Arabian context.

**Methods:**

A two-staged sequential mixed methods study was conducted among medical, dental, pharmacy, and health sciences students of the University of Sharjah United Arab Emirates. The perspectives of students toward IPE and collaborative practice were first gathered by administering a validated instrument, Readiness for Interprofessional Learning Scale (RIPLS). This was followed by focused group discussions. A quantitative as well as a qualitative data analysis was performed.

**Results:**

This study cohort included 282 students. All respondents showed readiness to adopt IPE as all statements of the RIPLS inventory scored high median scores. All participants showed positive attitudes and readiness towards IPE. Three main domains of themes were generated from focus group discussions; prior knowledge, need for IPE framework and its implementation. Information workload, lack of clarity and less focused teaching pedagogies of IPE were considered as perceived barriers.

**Conclusion:**

This study demonstrated a substantial agreement of medical and health sciences students towards readiness and perceived effectiveness of IPE. Educators are urged to embed new IPE programs into existing curricular frameworks, which can potentially enhance collaborative learning and improve quality of patient care.

**Supplementary Information:**

The online version contains supplementary material available at 10.1186/s12909-021-02645-4.

## Background

Historically, medical and allied health education have been delivered in an isolated educational environment. This uni-professional education limits exposure to collaborative learning, which is an essential element of Interprofessional Education (IPE) [1]. IPE entails certain opportunities where two or more professionals learn with, from and about each other [2]. This integrated approach is in sharp contrast to the multi-professional education that encourages health professionals to learn alongside each other in a parallel manner [3]. The outright benefits of IPE include promotion of interdisciplinary collaborative work [4], overcoming obstacles and misconceptions among healthcare groups and strengthening professional competencies [5]. Inter-professional practice engages healthcare professionals and multiple stakeholders such as patients, families and communities for improving the quality of patient care [6, 7]. From a different perspective, IPE fosters flattened team hierarchy with emphasis on a collaborative teamwork that ultimately secures patient safety by reducing errors [8]. Unfortunately, literature shows conflicting narratives and fails to offer a unified teaching framework that can be conveniently applied in achieving the desired goals of IPE philosophy [9]. In addition, whether IPE courses should be provided in the pre- or post-qualification phases, remains controversial [10, 11].

The IPE accreditation standards in the United States of America (USA) have urged all accrediting bodies to jointly collaborate for the development of a common IPE accreditation program [12]. In pursuit of further enhancement of IPE implementation in the medical field, the committee of Medical Education or “*Gesellschaft für medizinische Ausbildung* (GMA)”, including experts from Germany, Austria and Switzerland, has emphasized the need to systematically address and integrate IPE among other health professions [13]. Though in the last two decades, IPE has gathered momentum in the USA, Australia, and the United Kingdom (UK) [14], IPE is still in its infancy in the region of Middle East and North Africa (MENA) [15–18]. Recently, in an attempt to promote IPE, from the UK perspective, Courtenay et al., have proposed a protocol that can be designed to provide competencies for the national consensus on antimicrobial stewardship for undergraduate students [19]. Similarly, the Jefferson Teamwork Observation Guide® (JTOG®) was developed as a multi-source tool to formatively assess IPE and collaborative practice competencies [20]. Educating the learners about salient characteristics of high functioning teams has prompted the development of the JTOG® guide. In Japan, an interesting study investigated the impact of Japanese and Scottish experience of care of diabetes mellitus [21]. The investigators have found that the international standards of IPE set forth for this study were able to raise awareness of diabetes mellitus in terms of patient-centred focused care. In summary, globally there is a trend to inculcate a culture of multi-disciplinary collaboration and team-work.

The inter-woven and complex nature of instructional strategies in various healthcare disciplines hinders a smooth incorporation of IPE modules into the existing curricula [22]. Crowded timetables, logistical obligations requiring simultaneous movement of large numbers of students for undertaking similar classes, and lack of resources are some of the main challenges to IPE [23]. Freeth et al. have introduced a 5-point framework of IPE that is based on real-time scenarios, exchange of ideas, simulation, observation, and practice [24]. While such insights seem promising, before designing any program, educators must capture opinions and perceptions of key stakeholders such as students, faculty, administrators and community representatives. An analysis of needs and readiness for IPE curriculum is a first step in introducing a sustainable and relevant IPE program [25]. In the MENA region, the readiness and perspectives of medical and health sciences students about IPE are understudied. This is partly attributed to a limited introduction of IPE in the region and partly due to a system-based flaw that offers scarce opportunities for a collaborative interprofessional education and practice [26]. Additionally, an absence of an uninterrupted professional development IPE program with sustained planning, facilitation, and evaluation has hampered its acceptance and adaptation in the region [27]. Published reports have shown a positive attitudes of practicing physicians [28] and medical students [29] towards IPE, but unfortunately, there is limited data that can point out the challenges and hurdles for a smooth induction of IPE across medical curricula. Therefore, we have conducted this study to determine the readiness of undergraduate students enrolled in the medical and health sciences colleges towards IPE and to identify their perceived needs and challenges. This would be a stepping-stone towards the future adaptation of IPE in the MENA region and beyond. Consequently, educators would be able to effectively solicit and analyze such data for improving the learning climate and in drawing students’ motivation toward IPE.

## Materials and methods

### Research design

We adapted a mixed methods research design, which was conducted in quantitative and qualitative phases. The quantitative phase had a cross-sectional questionnaire-based research design, while the qualitative phase was based on focus group discussions. Students from the college of medicine (CoM), college of dental medicine (CDM), college of pharmacy (CoP), and the college health sciences (CHS) of the University of Sharjah (UoS) United Arab Emirates (UAE) participated in both phases.

### Study settings

The CoM supports a student-led learning philosophy and the 6-year program is based on problem based learning. At the CDM, a theme-based curriculum is employed. The college of CoP curriculum embraces diverse teaching approaches focusing on active learning, problem-based learning, and evidence-based learning. The CHS adopts different methods of teaching ranging from traditional lecturing to problem-based learning, team-based learning and small group discussions. The CHS includes a wide range of disciplines; nursing, physiotherapy, nutrition, diagnostic imaging, laboratory technology, environmental health and health service administration. At the time of conducting this study, there is no structured IPE course or module across all four medical and health science colleges of the UoS. However, there are limited IPE educational activities in the selected colleges but without a structured format.

### Phase 1: quantitative study

Students studying year four and five of the medical and allied health science colleges of the UoS were invited to participate in this study. At the end of a course lecture, students were informed about the purpose of the quantitative study and their verbal consent was sought. Students who voluntarily agreed to participate were asked to fill out an anonymous paper-based questionnaire. The questionnaire was divided into three parts. The first part inquired about some personal details including gender, age, college of enrollment and previous experience of IPE. The second part of the questionnaire included a validated scale, Readiness for Interprofessional Learning Scale (RIPLS) [30]. The scale contains 19 close-ended statements about the readiness of medical students for interprofessional education and practice [31]. The participants were instructed to respond on a 5-point-Likert scale in numerical values: 5 (strongly agree), 4 (agree), 3 (neutral), 2 (disagree), and 1 (strongly disagree) for all statements. In the third part of the questionnaire, students were asked to specify whether they were “with” or “against” IPE and whether they would be interested to participate in a focus group discussion to share their opinions about IPE. This research was conducted after obtaining ethical approval from the Research Ethics Committee at the UoS.

### Phase 2: qualitative study

During the quantitative phase, the students who expressed an interest to participate in a focus group discussion were invited to take part in the qualitative phase of the study. The students were verbally briefed about IPE and the nature of the study. The participants were instructed to respond whether they were in favor or against IPE and why.

The perceived attitudes of the respondents were context-based, which prompted the researchers to create focus groups for choosing appropriate group homogeneity. Exogenous homogeneity reflects shared group dynamics such as demographics or profession, while issue homogeneity denotes a shared response towards a particular issue. Consequently, we adopted issue homogeneity; grouping together students from different medical disciplines with similar attitudes that would encourage and facilitate self-disclosure. Therefore, students were grouped into two groups based on their attitude towards IPE; those who valued and those who did not. Data were collected until saturation was achieved. A total of four focus groups were conducted; two sessions for each group. Each focus group consisted of six to eight male and female students representing four colleges in the medical campus. Consent was obtained verbally during recruitment and upon participation in the focus group discussions. Each participant was given a participant information sheet (PIS) that includes all details pertaining to the research objectives and procedures, in addition to contact details of the study’s investigators and ethics committee in case s/he had any complaint or inquiry about the research. PIS is included in Additional file 1. Four faculty members with experience in focus groups moderated the discussions in separate private classrooms. There were two moderators in each focus group, one senior faculty member and another as a data collector and administrator. Subsequently, the data was analyzed by an independent researcher. Prior to the commencement of each session, moderators explained the ground rules and objectives of each session. All participants were briefed, verbally consented and then the PIS was handed over to the participants.

A set of unbiased and open-ended questions and probes were developed to elicit the following information from participants: experience with IPE, possible structure and implementation of IPE, and perceived advantages and disadvantages of IPE. All discussions were tape-recorded after taking verbal permission from the participating students.

### Statistical analysis

SPSS version 25.0 was used for data management and statistical analysis. Quantitative data were analyzed using descriptive statistics. Frequencies and percentages were reported for categorical data, while means and medians were reported for continuous data. Ordinal data obtained from individual items of the RIPLS scale were summarized using frequencies, percentages and medians. Responses on the 5-point Likert scale were recoded and clustered into three categories where responses were combined as strongly agree and agree, and disagree and strongly disagree. The internal consistency reliability of the RIPLS scale was measured using Cronbach Alpha. Four out of 19 items on the RIPLS scale were negatively worded and these were items 10, 11, 12 and 18. For example, item 10 states *“I don’t want to waste time learning with other health and medical students”*. Responses on the four negatively worded items were reversed where the code of 1 (strongly disagree) became 5 (strongly agree), 2 (disagree) became 4 (agree), 4 (agree) became 2 (disagree), 5 (strongly agree) became 1 (strongly disagree) while 3 (undecided) stayed unchanged. After reversing the negatively worded statements, a total score on the RIPLS scale was calculated by adding up students’ responses on the five-point scale. Similarly, total scores were calculated for the three subscales of the RIPLS questionnaire and these were subscale 1 (teamwork and collaboration), subscale 2 (professional identity) and subscale 3 (roles and responsibilities) [30]. Normality of the continuous data was visually tested using the Q-Q plots and statistically by Kolmogorov-Smirnov test. When parametric assumptions were met, the independent t-test and one-way ANOVA test were used to compare two or more than two means, respectively The non-parametric tests Mann-whitney U and Kruskal Wallis tests were used when the assumptions were not met statistically. A chi-square test was used to study the association between categorical variables and the level of significance set at 5%. Multiple linear regression analysis, using the stepwise method, was used to identify significant predictors of students’ preparedness for IPE. Criteria for inclusion of variables in the regression model was a *p*-value of 0.20 in the bivariate analysis.

Focus group discussion audio recordings were transcribed verbatim and de-identified. The six-stage thematic analysis framework by Braun & Clarke was used, which includes familiarization with the data, generation of initial codes, searching for themes, reviewing themes, defining and naming themes, and writing the final report [32]. We performed the initial coding of the transcripts and generated a coding tree. Key issues were identified and categorized for each focus group using deductive and inductive coding and the codes were grouped into themes and subthemes [33]. The deductive approach followed the previously defined themes (Experience with IPE, possible structure and implementation of IPE, and perceived advantages and disadvantages of IPE), while unique responses were coded inductively to create the categories.

### Emp*irical results*

Of the 300 invitees, 282 students participated in the quantitative phase of this study; a response rate of 94%. The majority of respondents were females aged between 20 and 24 years. Only 31 (11%) students reported to have a previous experience of IPE and a great majority (89%) were in favor of adapting IPE in the medical field. A detailed analysis of respondents’ profile is shown in Table 1.

**Table 1:** Profile of the study respondents (*N* = 282)

	n	%
Gender		
Male	45	15.9%
Female	237	84.0%
Age		
< 20	16	5.7%
20–24	251	89.0%
> 24	15	5.3%
College		
Health Sciences	93	32.9%
Dentistry	63	22.3%
Pharmacy	74	26.2%
Medicine	52	18.4%
Previous Experience of IPE		
Yes	31	10.9%
No	249	88.3%
Don’t know	2	0.7%

The reliability of the 19-item RIPLS scale was 0.687 as measured by Cronbach Alpha. More than 90% of students agreed that IPE makes them effective members in a healthcare team, increases their abilities to understand clinical problems, improves their collaborative practice and communication skills with other healthcare students and helps them to understand their own professional limitations. Moreover, 188 (66%) students disagreed or strongly disagreed that IPE is a waste of time, or IPE is unnecessary for undergraduate healthcare students, and that clinical problem solving skills can only be learned with students from their own field of study (Table 2).

**Table 2:** Descriptive results of the items of the Readiness for Interprofessional Learning Scale (RIPLS) scale as reported by students [*N* = 282; n (%)]

Statement	Strongly disagree/ disagree	Undecided	Agree/ strongly agree	Median
S1. Learning with other students will help me become a more effective member of a healthcare team.	5 (1.8%)	20 (7.1%)	257 (91.1%)	4.00
S2. Patients would ultimately benefit if healthcare students worked together to solve patient problems.	4 (1.4%)	9 (3.2%)	269 (95.4%)	5.00
S3. Shared learning with other healthcare students will increase my ability to understand clinical problems.	3 (1.1%)	16 (5.7%)	262 (92.9%)	5.00
S4. Learning between health and medical students before qualification would improve working relationships after qualification / collaborative practice.	5 (1.8%)	14 (5.0%)	262 (92.9%)	4.00
S5. Communication skills should be learned with other healthcare students.	6 (2.1%)	18 (6.4%)	258 (91.5%)	4.00
S6. Shared learning will help me to think positively about other professionals	8 (2.8%)	31 (11.0%)	240 (85.1%)	4.00
S7. For small-group learning to work, students need to trust and respect each other.	4 (1.4%)	21 (7.4%)	256 (90.8%)	5.00
S8. Team-working skills are essential for all healthcare students to learn.	4 (1.4%)	30 (10.6%)	248 (87.9%)	4.00
S9. Shared learning will help me to understand my own professional limitations	2 (0.7%)	10 (3.5%)	270 (95.7%)	4.00
S10. I do not want to waste my time learning with other healthcare students.	194 (68.8%)	51 (18.1%)	35 (12.4%)	2.00
S11. It is not necessary for undergraduate healthcare students to learn together.	191 (67.7%)	54 (19.1%)	35 (12.4%)	2.00
S12. Clinical problem-solving skills can only be learned with students from my own department.	158 (56.0%)	50 (17.7%)	69 (24.5%)	2.00
S13. Shared learning with other healthcare students will help me to communicate better with patients and other professionals.	18 (6.4%)	23 (8.2%)	235 (83.3%)	4.00
S14. I would welcome the opportunity to work on small-group projects with other healthcare students.	13 (4.6%)	37 (13.1%)	229 (81.2%)	4.00
S15. Shared learning will help to clarify the nature of patient problems.	13 (4.6%)	41 (14.5%)	225 (79.8%)	4.00
S16. Shared learning before qualification will help me become a better team worker	7 (2.5%)	29 (10.3%)	244 (86.5%)	4.00
S17. I would welcome the opportunity to share some generic lectures, tutorials or workshops with other health and medical students	11 (3.9%)	24 (8.5%)	240 (85.1%)	4.00
S18. I am not sure what my professional role will be / is	125 (44.3%)	94 (33.3%)	61 (21.6%)	3.00
S19. I have to acquire much more knowledge and skill than other students in my own college	43 (15.2%)	89 (31.6%)	148 (52.5%)	4.00

**Fig. 1:**
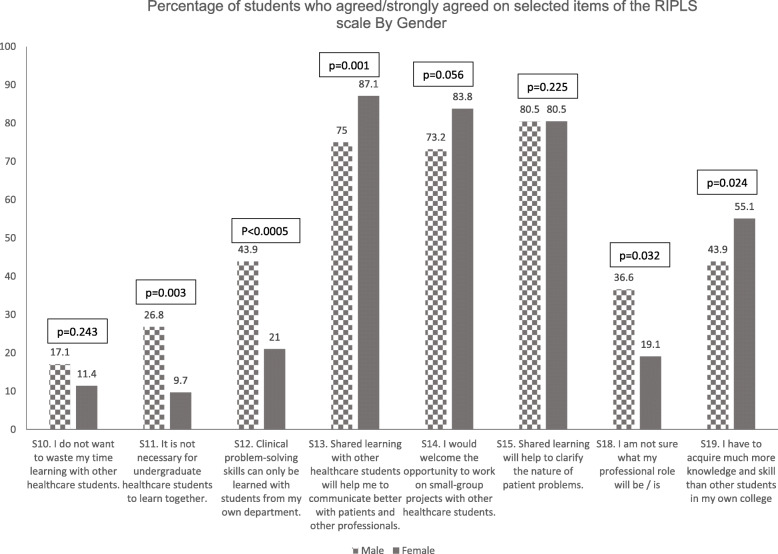
Percentage of students who agreed/strongly agreed on selected items of the RIPLS scale By Gender

Male students agreed with the statements *“It is not necessary for undergraduate healthcare students to learn together”*, *“Clinical problem solving skills can only be learned with students from my own department”*, and *“I am not sure what my professional role will be/is”*. On the other hand, more females than males agreed that *“Shared learning with other healthcare students will help me to communicate better with patients and other professionals”* (Fig. 1).

**Fig. 2:**
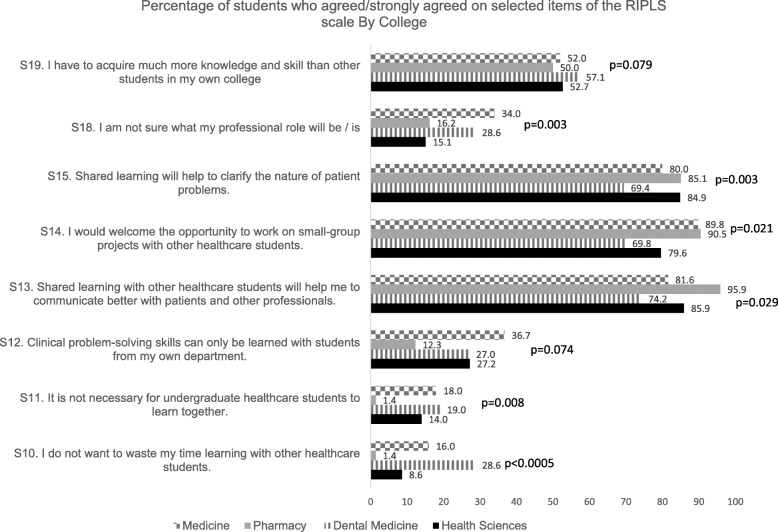
Percentage of students who agreed/strongly agreed on selected items of the RIPLS scale By College

The responses of the majority of students were significantly associated with statements S10–15, S18 and S19 on the RIPLS scale (Fig. 2).

The students’ RIPLS total scores ranged between 44 and 95 where the median score was 78. Bivariate analyses revealed that the total and subscale RIPLS scores were significantly related to their attitude towards IPE. Scores of students who reported being with IPE were significantly higher than those who were against IPE. Pharmacy students had significantly higher scores on the RIPLS total and three subscales than all students from other three colleges. Professional identity and roles and responsibilities subscales scores were significantly higher for female students as compared to males. Scores on the roles and responsibilities subscale were higher for students with previous IPE experience compared to those who did not have a prior IPE experience (Table 3).

**Table 3:** Students scores to the RIPLS for overall and for subscales of gender, college, and experience of interprofessional education (*N* = 282)

	RIPLS total score			Subscale 1 – Teamwork and collaboration			Subscale 2 – Professional identity			Subscale 3 – Roles and responsibilities		
	Median	Test-statistic^**a**^	***p***-value	Median	Test-statistic^**a**^	***p***-value	Median	Test-statistic^**a**^	***p***-value	Median	Test-statistic^**a**^	***p***-value
Gender												
Male	75.0	3666.0	0.118	40.5	4794.5	0.904	25.0	3280.0	0.008	10.0	3801.0	0.025
Female	78.0			40.0			28.0			11.0		
College												
Health Sciences	77.0	18.016	< 0.00 05	40.0	10.458	0.015	28.0	23.111	< 0.0005	11.0	10.572	0.014
Dental Medicine	75.0			39.5			26.0			10.0		
Pharmacy	80.5			41.0			29.5			11.0		
Medicine	75.5			38.0			27.0			11.0		
Previous experience with IPE												
No	77.0	2872.	0.0	40.0	336	0.33	28.0	3375.	0.43	11.0	2471.	0.0
Yes	80.0	5	74	41.0	4.5	1	28.0	0	9	12.0	5	01
Attitude towards IPE	78.0	569.5	< 0.00 05	41.0	742.5	< 0.0005	28.0	598.0	< 0.0005	11.0	848.5	0.0
With Against	65.0			34.0			21.0			10.0		05

^a^test-statistic is Mann-whitney U test and Kruskal Wallis test for gender and Previous experience with IPE and Kruskal Wallis for College

A regression analysis was done to predict students’ RIPLS score using four factors of gender, college, experience with IPE and attitude towards IPE. The regression model was significant in predicting 21.6% of the total variability of the dependent variable [F (1, 250) = 70.302, *p*-value < 0.0005]. Using the stepwise method, the regression analysis showed that students’ attitude towards IPE was the only significant predictor of the RIPLS score (beta = − 14.255, *p*-value< 0.0005). Therefore, being against IPE, as compared to being with IPE, is expected to significantly reduce a student’s RIPLS score by 14 points.

### Qualitative results

**Fig. 3:**
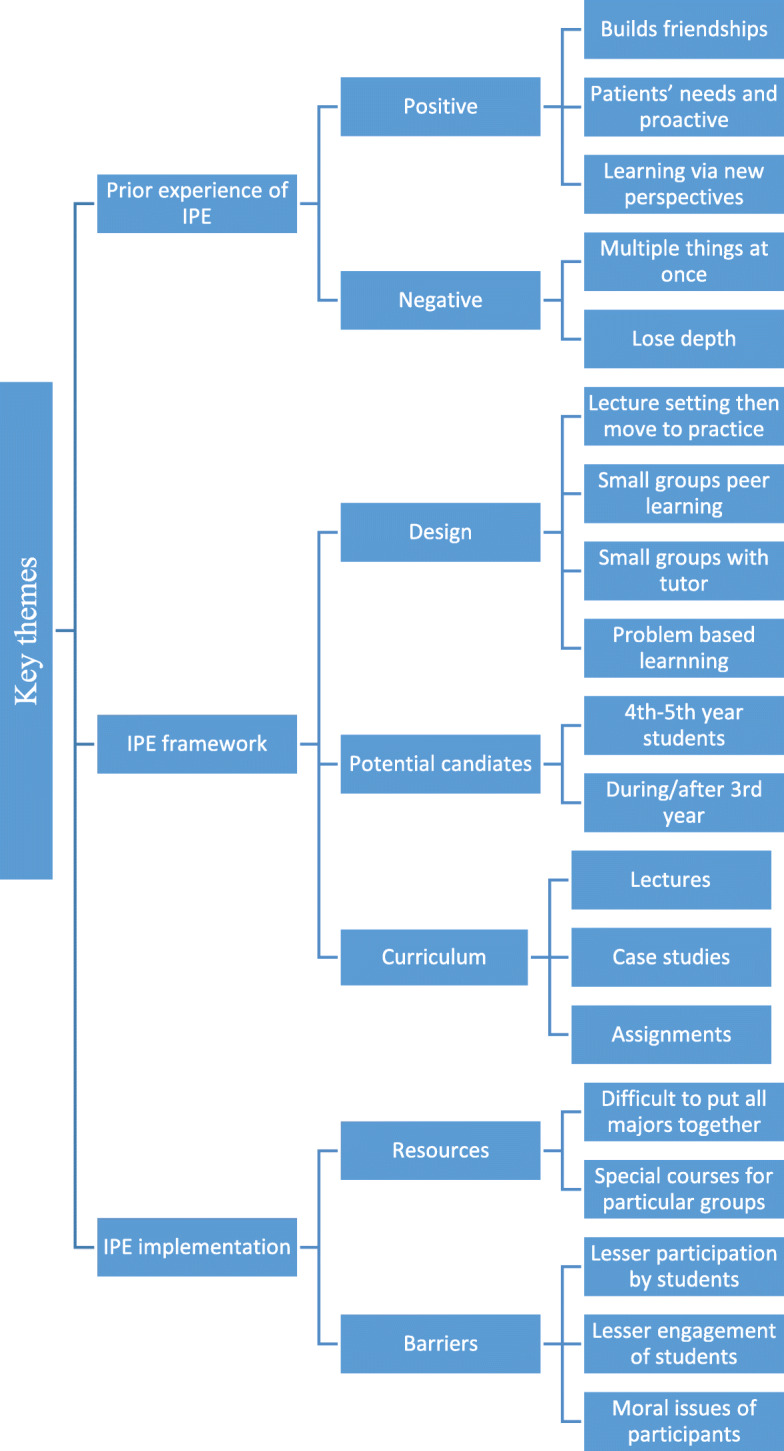
Qualitative analysis of open questions using the NVIVO with identified themes

In this study, we identified three broad domains; prior knowledge of IPE, framework for IPE and implementation of IPE (Fig. 3).

### Experiences

As reflected from the recorded quotes during focus group discussions, students provided opinions about IPE positive and negative experiences. Students’ quotes describing their positive experiences were clustered around three themes; building friendships, fulfilling patients’ needs, and learning from different perspectives. Furthermore, students’ negative experiences were classified into two themes; learning multiple things at once and losing depth in one’s discipline. An interpretation of each theme under the positive and negative experiences as reflected by students during the focus group discussions is outlines hereunder.

### Positive experiences

#### Building friendships

Learning with peers enrolled in other colleges and departments was highly valued by students. They appreciated the opportunity to meet students from different majors and building friendships with peers. Students appreciated IPE courses as an opportunity to promote their social life in the university. Below are examples of students’ quotes supporting this theme:

*“During first year of foundation of sciences, we were studying physics and chemistry and biology with other specialists, such as medical diagnostic imaging and nutrition. We were studying together; it was so fun we didn't differentiate between others”.*

“*We know more about how our friends are, and how we built friendships.”*

#### Fulfilling patients’ needs

Students perceived IPE as a means of promoting their holistic approach when managing a patient. A group of students from different specialties working on various perspectives of a patient’s health problem resulted in a comprehensive management plan. This strategy meets patient’s expectations and needs. Following is an example of students’ quotes relevant to this theme.

*“Comprehensive and overlapping treatment plans allow us to not only look at patients as diseases but allow us to look at all their needs and prevent future problems from happening. So, if we do this with more peers from other majors, it will actually drive us to look and help the patient in more than just his chief complaint. IPE will allow us to look at everything and prevent future problems from happening”.*

#### Learning from different perspectives

Students perceived IPE as a platform which encouraged them in broadening and diversifying their problems solving approach. Students valued the importance of benefiting from each other’s knowledge and expertise in order to reach a collective decision in patient care. Working with students from other disciplines helped students to bridge gaps in their knowledge about patient care. The following quotes support this theme.

*“Basically, we share different experiences and different ways of thinking, for example, for me in Pharmacy, we think about medicines, just the drugs used. In Nursing, they think about the patients more regardless of their medical problems. In Medicine, they think about diseases so everyone thinks in different ways so the way we act or react is different, so we are learning from each other’s perspectives.”*

*“I believe it should be mostly problem based learning so that when we get a piece of information, we do not only look at it from one perspective, but we have Pharmacy, Dentistry and so on. There they have different points of views that we may not consider and won’t come to our mind at all.”*

### Negative experiences

#### Learning multiple things at once

Besides the benefits of IPE, students found IPE courses to be sometimes burdensome. They found it challenging to focus on multiple perspectives of a problem simultaneously. One example of students’ quotes is the following:

*“Because we are focusing on multiple things at once but it wastes a lot of time while framing a holistic view, for example, for taking pharmacology of stroke or cardiovascular, if we are taking that at the same time, who would need it more.”*

#### Losing depth in one’s discipline

One of the negative experiences emerging from IPE is losing depth in one’s own discipline. Students felt that they needed to focus on their specific areas of specialization and not to waste their time in understanding other disciplines. They argued that IPE was associated with a certain level of distraction from one’s own discipline such as:

*“You lose depth when you are studying although you would be gaining depth in other people's majors.”*

**Fig. 4:**
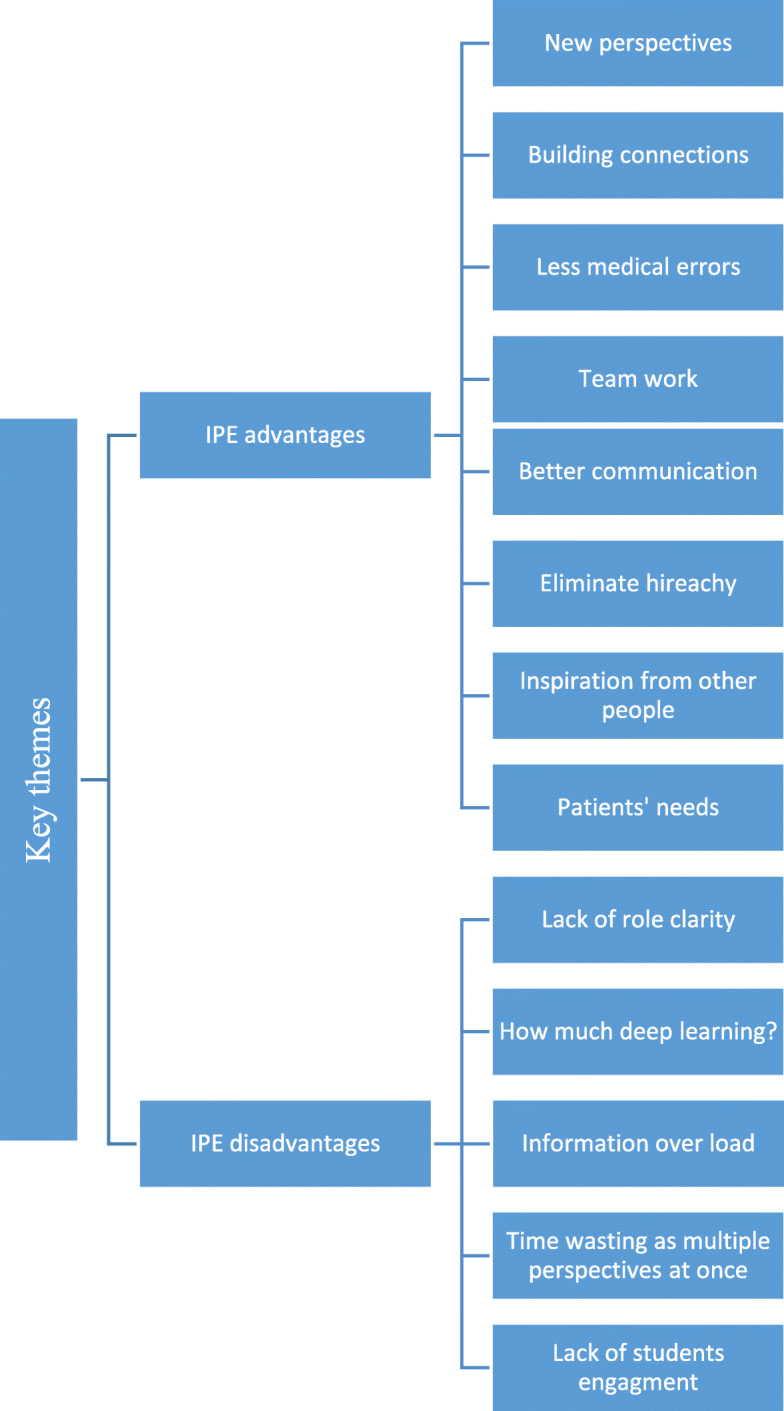
The perceived advantages and disadvantages of interprofessional education in medical field by participants of this study

A detailed layout of the qualitative analysis from all respondents is shown in Fig. 4.

## Discussion

This study illustrates a strong agreement of the participants about readiness for IPE as well as positive attitudes to implement this insightful educational model into the medical and health sciences curricula. The findings of our research endorse previous reports that validate the readiness to accept IPE program [34, 35]. The presence of positive attitude towards IPE signifies a clear understanding and mandates the incorporation of IPE initiatives within institutional frameworks. Kapur et al., have reaffirmed that collaborative discussion and sharing of information offer the learners a unique chance for reflection and empower them to take crucial decisions [36]. The educational climate including IPE enlightens learning experience of the students that encourages them to respect and recognize roles and responsibilities amongst team members [37]. This approach certainly enables teamwork and collaboration with a positive effect on the quality of patient care.

This study demonstrates substantial agreement by the respondents for IPE particularly for statements 2 and 3, which illustrate significance of IPE in understanding and solving patients’ problems. This reiterates the valuable role of IPE and practice in managing a host of medical ailments when healthcare professionals from various disciplines join their hands together in the medical field. By practicing an interprofessional teamwork, not only responsibilities are shared, but also the changes of medical errors are minimized [38]. Bartaw et al., have argued that a standardized approach by a specialized and multidisciplinary team can substantially reduce the incidence of complications and ends up with better patient care outcomes [39]. Our study cohort has also shown the highest agreement with the positive influence of IPE in small group learning that helps to enhance trust and respect among the learners. Small group learning has been shown to enhance the acquisition of knowledge and professional skills of the students that leads to active life-long learning [40]. Interestingly, Laal and Ghodsi have introduced four major benefits of small group learning; social (inspirational environment for practicing cooperation), psychological (reduces stress and increases learner’s self-esteem), academic (improves academic performance and critical thinking skills), and assessment (applying diverse assessment techniques for holistic assessment) [41]. However, the authors have cautioned that such milestones need expertise and a positive attitude towards implementing IPE program in medical curricula.

Our study has identified three broad educational domains of IPE; prior knowledge, framework and implementation of IPE. In their review article, Hall and Zierler have provided a framework for developing and implementing IPE program [42]. To start with, the authors have suggested to secure a commitment by institutional leadership, followed by drafting context-based learning objectives. Then a well-structured faculty development program would train the academia for IPE. The authors have concluded that the outcomes should be carefully measured during the process of implementing IPE and educators should be able to establish robust links between theory and practice. Employing diverse teaching pedagogies such as lecturing, small group work, immersion participation in IPE, embedding new IPE projects, and peer-assisted learning are some of the suggested educational strategies in this context. Working on a qualitative research, Sanko et al., have reported main themes; articulated learning, recognition of other colleagues’ opinions and identification of one’s deficiencies in knowledge and skills [43]. The authors have presented IPE as a platform that can be used for a comprehensive collaborative teamwork in the medical field. Another report has shown that the IPE program was able to encourage genuine engagement and the students were able to benefit from the opportunities to interact with students from other professions [44]. Thus, by and large, the arena of IPE in the medical field is well accepted, but lacks a structured program that can be applied globally.

Unfortunately, at the time of conducting this study, a number of practicing health professionals have little or no exposure to IPE exercises during their training. Consequently, faculty development program as well as work-place based education using technology are vital training tools that can facilitate successful embedding of new IPE module for effective teaching and learning [45, 46]. Key benefits that emerged from our study during focus group discussions include better communication, encouragement from peers, and shared decision-making. However, a lack of role clarity, information overload and less focused teaching strategies have been shown to be disadvantageous in IPE process. In terms of positive perspectives of IPE, our study cohort has agreed on building friendships, patients’ needs and proactive and active learning. These findings reinforce the perception that IPE strengthens professional ties [47], helps to understand and resolve patient’s problems and facilitates active learning [48]. In contrast, our cohort has also signaled some negative aspects of IPE such as multiple things at once and losing depth. Competing interests from other professions, inclination of learners to learn more from their major topics and multi-tasking have been shown to undermine true essence of IPE practice [49, 50]. From educators’ perspectives, embedding a new IPE module into the existing curricula and increasing faculty workload also challenge a smooth induction of IPE program. Provision of adequate resources, rescheduling faculty time, institutional support by focused IPE faculty development program [51] and horizontal and vertical induction of IPE modules into the curriculum can overcome these shortcomings [52].

### Study limitations

This study provides a comprehensive account of students’ perceptions of IPE with a reasonably high acceptability rate to RIPLS survey as well as by focus group discussion. This provides a substantial insight into the opinions and viewpoints of the students toward IPE. Nevertheless, since the findings of this study are self-reported perceptions and attitudes, the results cannot be interpreted in a context-based situation. Furthermore, since the majority of the recruited population were female students, external validity of this research might have been compromised. Lastly, our study represents data from a single-stage investigation. More longitudinal studies are essential for a reliable and reproducible analysis of results.

## Conclusion

This study provides evidence about the readiness of medical and health sciences students for IPE in a gulf university. A great majority of the students showed positive attitudes and readiness to adopt IPE. The students agreed about the effective role of IPE in collaborative work, in identifying and resolving patients’ problems and in minimizing medical errors. However, students pointed out some challenges; information overload, lack of clarity and unnecessary competition. A carefully planned faculty development program, engaging institutional leadership, a vertical and horizontal integration of new IPE courses and institutional support can potentially facilitate its seamless integration.

## Supplementary Information

**Additional file 1.**

## Data Availability

The SPSS raw dataset is provided. Additional data can be provided on request. The corresponding author, Salman Yousuf Guraya, will provide additional data, if requested.

## Abbreviations

CHS: College of Health Sciences
CoM: College of Medicine
CoP: College of Pharmacy
DOCEE: Direct Observation of Clinical Examination Encounter
GMA: Gesellschaft für medizinische Ausbildung
IPE: Interprofessional Education
JTOG®: Jefferson Teamwork Observation Guide®
MCQs: multiple choice questions
MDI: Medical Diagnostic Imaging
MENA: Middle East and North Africa
MLS: Medical Laboratory Sciences
OSCE: Objective Structured Clinical Examination
OSPE: Objective Structured Practical Examination
RIPLS: Readiness for Interprofessional Learning Scale
SPSS: Statistical Package for the Social Sciences
