# Supplementary material for: A mixed methods approach to determine the climate of interprofessional education among medical and health sciences students
Source: BMC Med Educ. 2021 Apr 10;21:203. doi: 10.1186/s12909-021-02645-4 (PMC8035734; doi:10.1186/s12909-021-02645-4)
Supplement: Supplementary file 1 — Additional file 1. [file 12909_2021_2645_MOESM1_ESM.docx]

**Additional file 1**

**Participant Information Sheet**

**Research Title:** Interprofessional Education: Impressions and Readiness of students in the Medical and Health Sciences campus of University of Sharjah

You are invited to be part of this study about UOS students’ ideas and opinion about Interprofessional Education (IPE), which is when undergraduate students from different health professions or majors (Pharmacy, Medicine, Health Sciences and Dentistry) learn together, about and from each other in a collaborative environment.

The purpose of this study is to explore the students’ thoughts and opinions about IPE, their perceptions of its advantages and/or disadvantages, as well as their readiness for potential implementation into the curriculum.

Participating in the study is completely optional and on a volunteering basis.
The study involves focus group discussions, which are group interviews were volunteering participants are asked questions and prompted to express their thoughts and ideas about the topic of discussion. There are mixed opinions about IPE, some are with and some are against the concept. Both of these stances are very important to the study. Should you choose to participate, you will be assigned into separate groups depending on whether you are with IPE or against it.
A moderator/facilitator will lead the discussion by initiating, maintaining and directing the conversation.
The focus groups will be conducted in private closed classrooms in the Medical College Building (M27).
The discussion will be recorded using an audio recording device for later analysis. All data will be confidential in that no participant names will be mentioned during the interviews, and the recorded data will be confidentially stored with the researchers.
Should you choose to participate, you will be attending a single focus group session, which shall take no longer than 90 minutes.

Participants will be acknowledged section in the publication if they choose to disclose of their identity and participation in the study.

For any questions or further information, please contact any of our team of researchers:
Prof Nabil Sulaiman, [nsulaiman@sharjah.ac.ae](mailto:nsulaiman@sharjah.ac.ae)

Dr Hamzah AlZubaidi, [halzubaidi@sharjah.ac.ae](mailto:halzubaidi@sharjah.ac.ae)

Dr Maha Saber, [msaber@sharjah.ac.ae](mailto:msaber@sharjah.ac.ae)

Prof Sausan Al Kawas, [sausan@sharjah.ac.ae](mailto:sausan@sharjah.ac.ae)

Dr. Youssef Rishmawy, [yrishmawy@sharjah.ac.ae](mailto:yrishmawy@sharjah.ac.ae)

Dr. Haydar Hasan, [haidarah@sharjah.ac.ae](mailto:haidarah@sharjah.ac.ae)

For any concerns, complaints or doubts, you may also contact the Research Ethics Committee at [rec@sharjah.ac.ae](mailto:rec@sharjah.ac.ae).
